# Concurrent Sedative Dosing Risks in Older Adults: Evidence from a National EMS Sample

**DOI:** 10.1093/geroni/igaf122.3737

**Published:** 2025-12-31

**Authors:** David Hancock, Rana Barghout, Josh Lachs, Tony Rosen

**Affiliations:** Weill Cornell Medicine / NewYork-Presbyterian Hospital, New York, New York, United States; Weill Cornell Medicine / NewYork-Presbyterian Hospital, New York, New York, United States; Weill Cornell Medicine, New York, New York, United States; Weill Cornell Medicine / NewYork-Presbyterian Hospital, New York, New York, United States

## Abstract

The rapid control of agitation in older adults often requires sedatives. Despite the heightened vulnerability in this group, little is known about how multiple medications are combined to address agitation in prehospital care. We analyzed national EMS data to characterize both timing and dosing patterns of sedative administration among patients ≥65 years (N = 3,132) who received more than one sedative medication during emergency responses. Ketamine-midazolam was the most common combination (61.7% of cases), followed by haloperidol-midazolam (18.4%). Ketamine-midazolam showed moderate concurrent use (39.5% within five minutes), with ketamine typically administered first when medications were staggered. Most haloperidol-midazolam administrations occurred within five minutes of each other (66.1%), suggesting concurrent use that maximizes sedation and potentially compounds age-related pharmacokinetic changes. By contrast, lorazepam-based combinations more often followed a sequential pattern, with more than five minutes between administrations. These differences are clinically meaningful: concurrent administration of sedatives in older adults presents elevated risk for oversedation, respiratory depression, and delirium, complications that disproportionately affect this vulnerable population and can trigger severe adverse outcomes. Staggered use of lorazepam combinations aligns more closely with geriatric emergency medicine recommendations to “start low and go slow.” However, across all combinations, the median doses administered to older adults were consistently above geriatric dosing protocols recommended for emergency settings, instead mirroring prescribing patterns used in younger adults. These findings underscore the need for age-specific prehospital protocols that balance the immediacy of behavioral emergencies with medication safety, ensuring strategies reflect the vulnerability of older adults to adverse sedative effects.

